# Unusual presentation of rare primary lymphoma of bone

**DOI:** 10.11604/pamj.2015.22.317.8232

**Published:** 2015-12-02

**Authors:** Malala Razakanaivo, Nomeharisoa Rodrigue Emile Hasiniatsy

**Affiliations:** 1 Oncology Unit, University Hospital of Antananarivo, Hôpital Joseph Ravoahangy Andrianavalona, Antananarivo, Madagascar; 2 Medical Oncology Unit, Centre Hospitalier de Soavinandriana, Antananarivo, Madagascar

**Keywords:** Bone, lymphoma, imaging

## Image in medicine

Primary bone lymphoma is a distinct disease. It represents only 3% of all malignant bone tumours and less than 1% of non-Hodgkin's lymphoma. It is essential to differentiate it from other tumours because of its good prognosis. We report a case of 45 years old male who presented one year ago with a painful left arm. His symptoms started after minimal trauma. The pain persisted for 6 months and became severe. It was managed by traditional medicine a practitioners who diagnosed it as a shoulder's sprain and did several massages sessions. The patient had worsening of his pain. On physical examination, shoulder movements were painful. Left arm was entirely swell. Superficial lymph nodes were not enlarged. The X-ray radiograph of the left upper extremity showed a fracture of the proximal humerus and large condensation lesion of the all humerus. Patient was posted for an open biopsy which revealed a primary diffuse large B cell lymphoma. Flow cytometric immunophenotyping showed positivity for CD10, CD20 and BCL6 and negativity for CD5, MUM1 and BCL2. Bone marrow and cerebrospinal examinations were normal. Contrast enhanced computed tomography of chest and abdomen failed to show evidence of any lymphomatous deposits. The patient was treated with chemotherapy including cyclophosphamide, doxorubicin, vincristine, and prednisolone with a good clinical response. He has been lost to follow up after five cycles of chemotherapy because of financial problem. The diagnostic study should begin with simple X-ray but adequate biopsies for histologic examination remain the standard in diagnosis.

**Figure 1:**
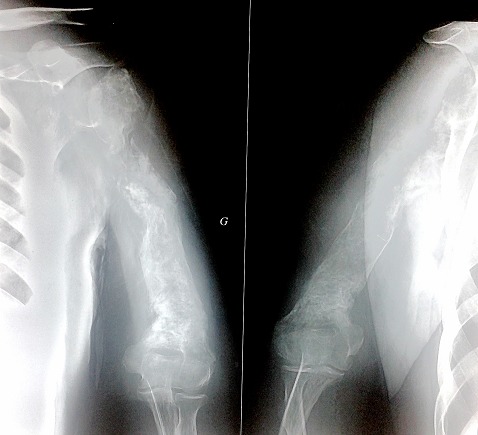
Unusual presentation of rare primary lymphoma of bone

